# Supplementary material for: High light and temperature reduce photosynthetic efficiency through different mechanisms in the C4 model Setaria viridis
Source: Commun Biol. 2021 Sep 16;4:1092. doi: 10.1038/s42003-021-02576-2 (PMC8446033; doi:10.1038/s42003-021-02576-2)
Supplement: Supplementary file 2 — Supplementary information. [file 42003_2021_2576_MOESM2_ESM.pdf]

High Light and Temperature Reduce Photosynthetic Efficiency through Different Mechanisms in the C_4 Model *Setaria viridis*

Cheyenne M. Anderson[1#], Erin M. Mattoon[1,2#], Ningning Zhang[1], Eric Becker[1], William McHargue[1], Jiani Yang[1], Dhruv Patel[3], Oliver Dautermann[3], Scott A. M. McAdam[4], Tonantzin Tarin[5,6], Sunita Pathak[1], Tom J. Avenson[7], Jeffrey Berry[1], Maxwell Braud[1], Krishna K. Niyogi[3,8,9], Margaret Wilson[1], Dmitri A. Nusinow[1], Rodrigo Vargas[5], Kirk J. Czymmek[1], Andrea L. Eveland[1], Ru Zhang[1*]

[#] Equal contribution

Supplementary Figures

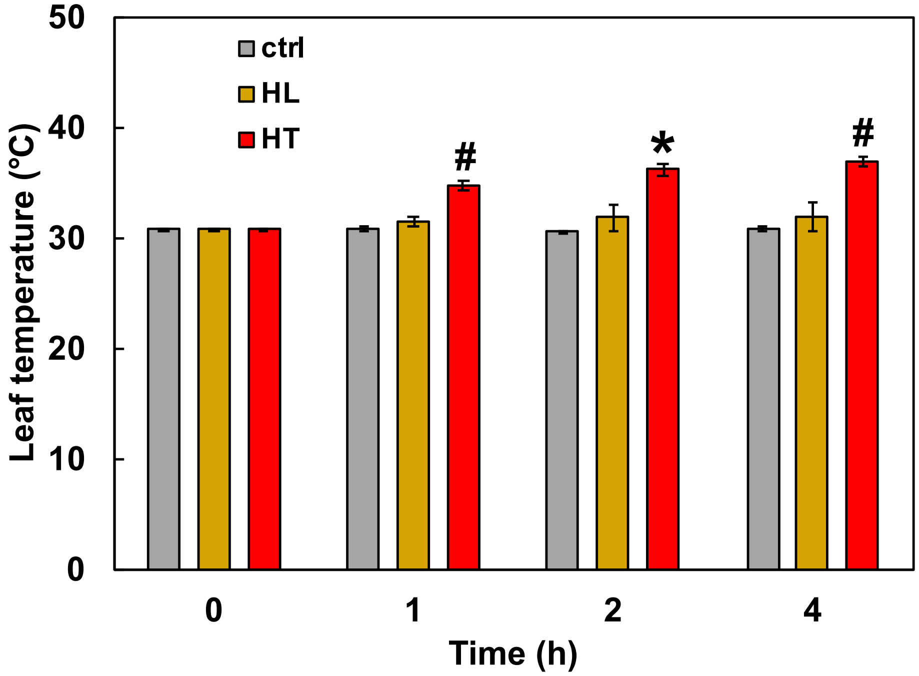

Supplementary Figure 1. Leaf temperatures of *S. viridis* stayed constant during the control and high light treatments while increased during high temperature treatment. Leaf temperatures of *S. viridis* measured over the 4 h time course of control or high light or high temperature treatments. Mean ± SE, n = 3 biological replicates. Asterisk and pound symbols indicate statistically significant differences of high light and high temperature compared to control in a given time point using Student's two-tailed t-test with unequal variance (*0.01<p<0.05, #p<0.01). No significant changes of leaf temperatures during the ctrl and high light condition.

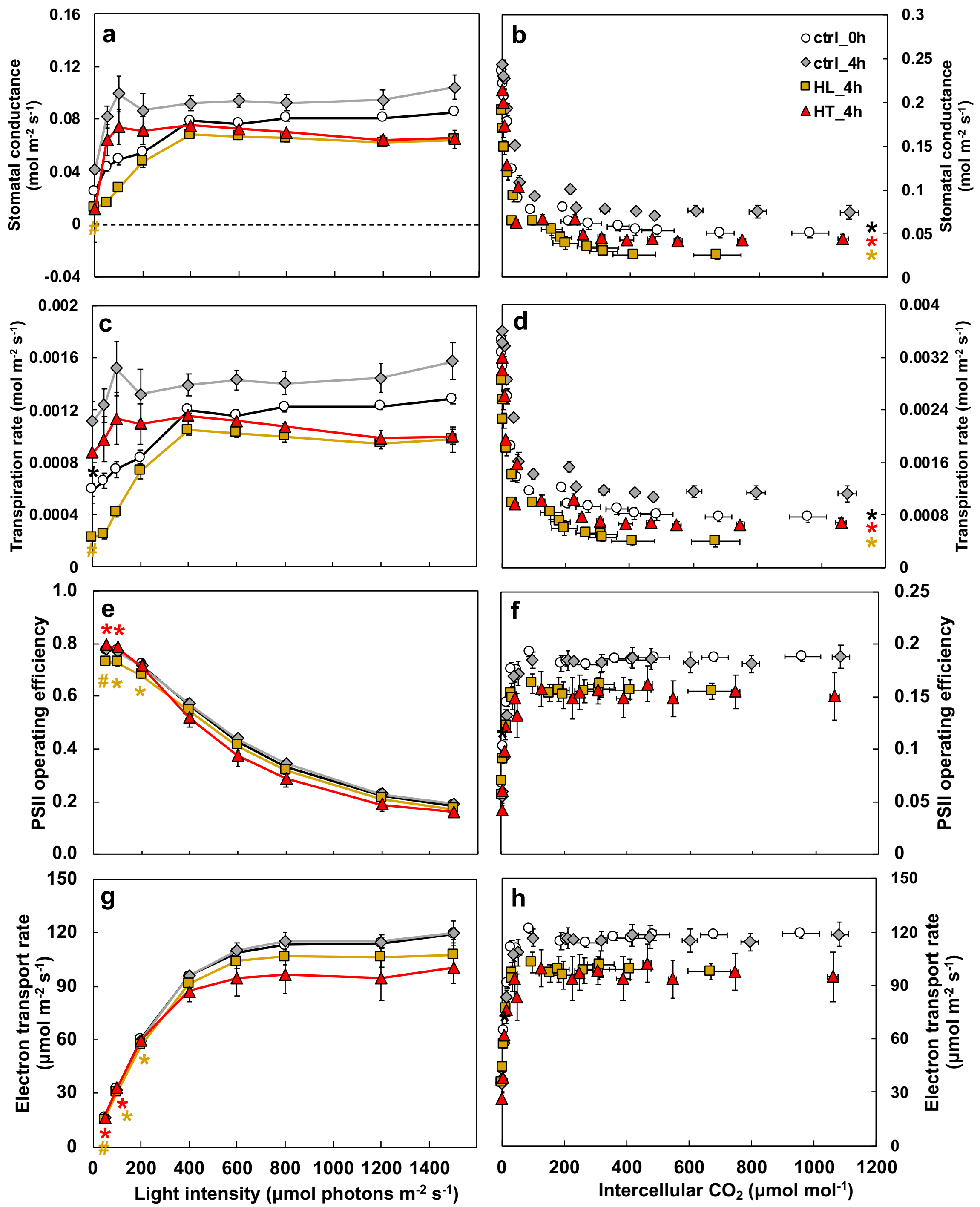

Supplementary Figure 2: High light or high temperature treatments affected photosynthetic parameters measured by gas exchange and chlorophyll fluorescence. Photosynthetic parameters measured during light **(a, c, e, g)** and CO_2 **(b, d, f, h)** response. Mean ± SE, *n* = 3-6 biological replicates. Asterisk and pound symbols

indicate statistically significant differences of ctrl_0h, HL_4h, and HT_4h compared to ctrl_4h using Student's two-tailed t-test with unequal variance. P-values were corrected for multiple comparisons using FDR (*0.01<p<0.05, #p< 0.01, the colors of * and # match the significance of the indicated conditions, black for ctrl_0h, yellow for HL_4h, red for HT_4h). (**b, d**) Most data points of ctrl_0h, HL_4h, and HT_4h were statistically significantly different compared to ctrl_4h (*p<0.05) denoted by asterisks at the end of curves.

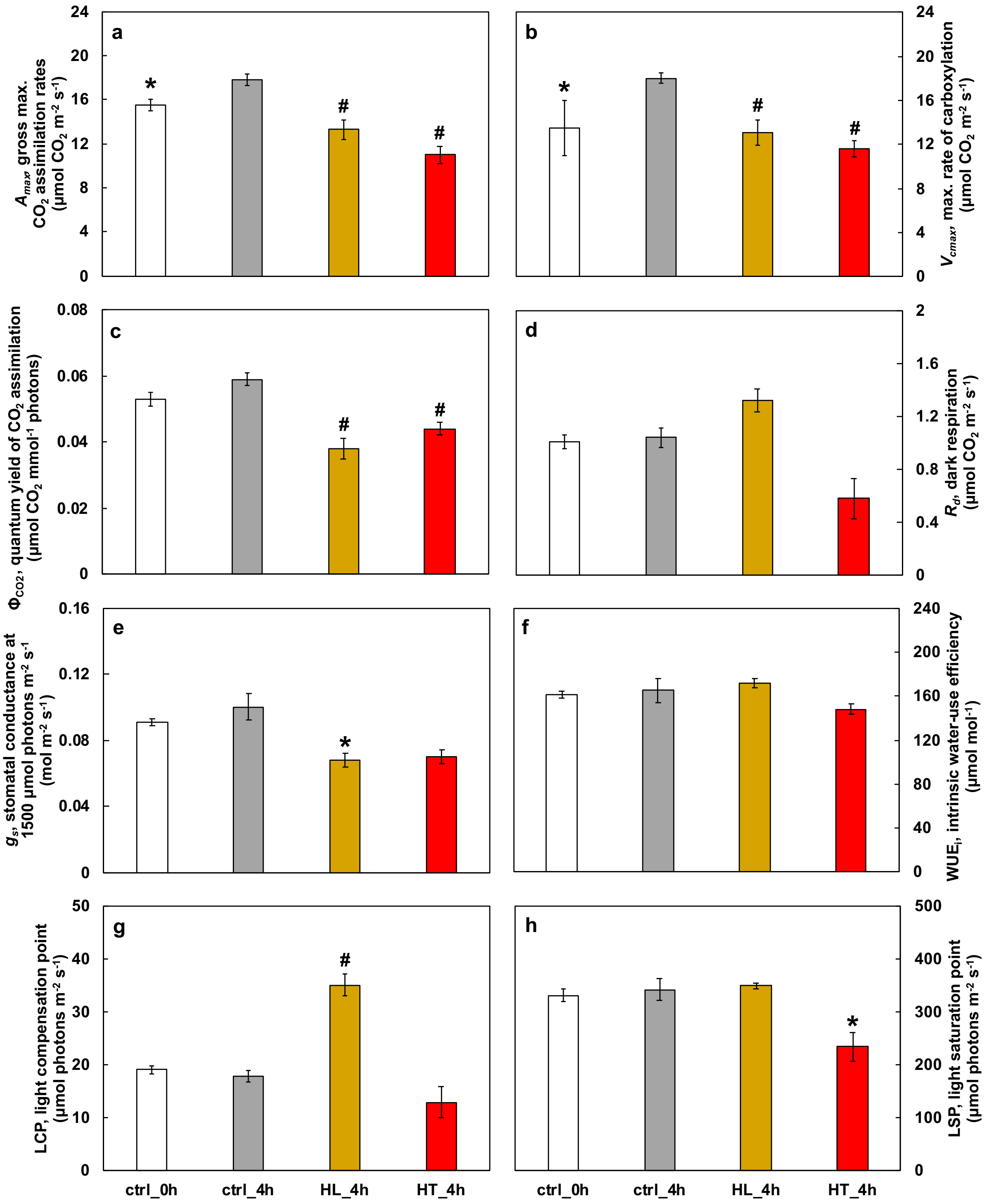

Supplementary Figure 3: High light or high temperature treated leaves had reduced photosynthetic efficiency. Photosynthetic parameters were derived from light and CO_2 response curves (mean ± SE; n = 3-6). **(a)** the maximum gross CO_2 assimilation rates, A_{max}; **(b)** the maximum rate of carboxylation, V_{cmax}; **(c)** the quantum yield of CO_2

assimilation, Φ_{CO2}, which is the ratio of the moles of CO_2 fixed in photosynthesis per mole of quanta (photons of light) absorbed, and is a measure of the efficiency in which light is converted into fixed carbon; **(d)** the day-time dark respiration rate, R_d, equal to A_n when light intensity is zero; **(e)** stomatal conductance, g_s; **(f)** water use efficiency, WUE; **(g)** light compensation point, LCP, the threshold of low light intensity at which photosynthesis is equal to leaf respiration and, therefore A_n is zero; **(h)** light saturation point, LSP, the estimated light intensity where 75% of A_{max} was reached. Asterisk and pound symbols indicate statistically significant differences of ctrl_0h (at the start of treatments), HL_4h (after 4 h high light), and HT_4h (after 4 h temperature) compared to ctrl_4h (after 4 h control treatment) using Student's two-tailed t-test with unequal variance (*0.01<p<0.05, #p< 0.01).

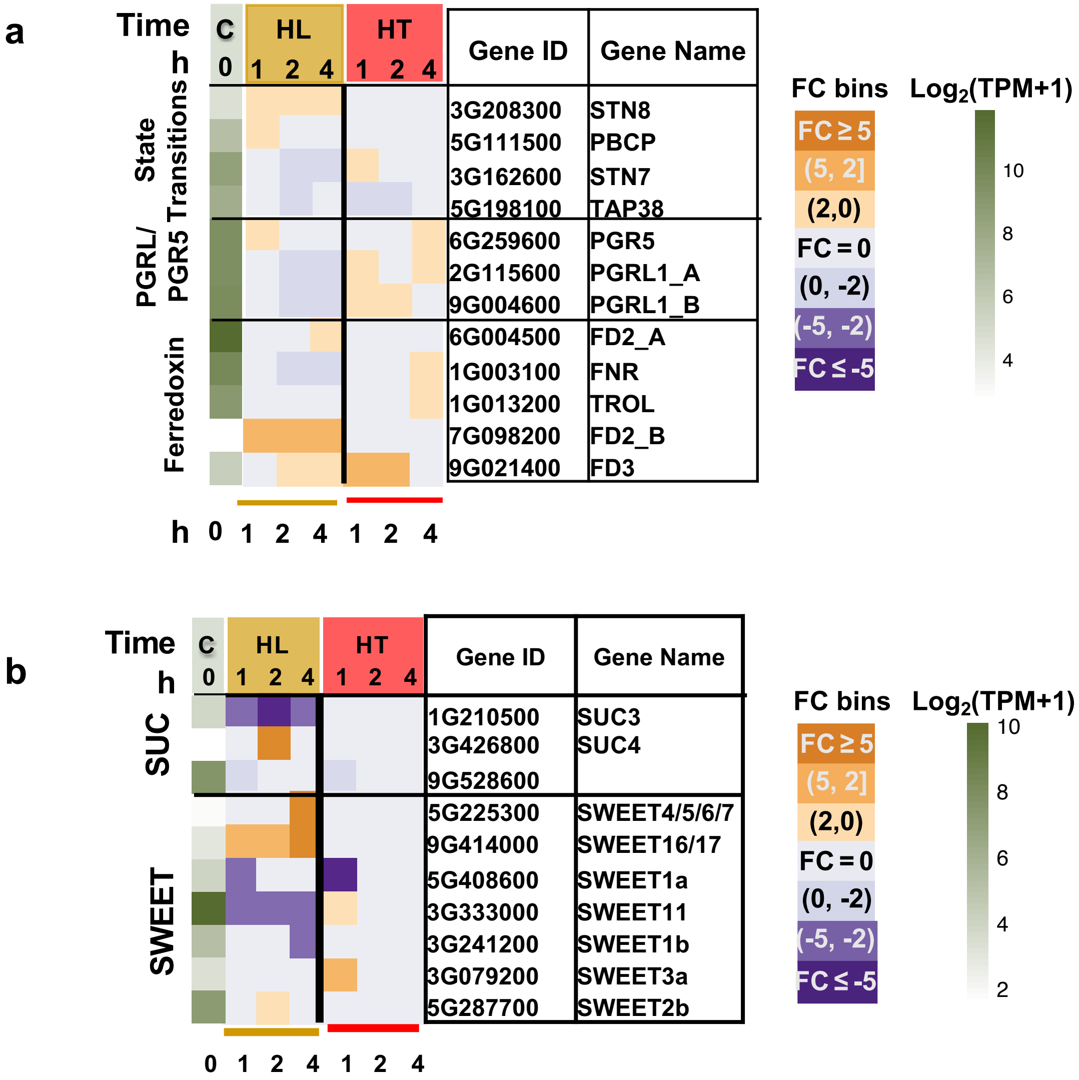
a
Time
h
C
0
HL
1 2 4
HT
1 2 4
Gene ID
Gene Name
State Transitions
PGRL/ PGR5
Ferredoxin
3G208300 STN8
5G111500 PBCP
3G162600 STN7
5G198100 TAP38
6G259600 PGR5
2G115600 PGRL1_A
9G004600 PGRL1_B
6G004500 FD2_A
1G003100 FNR
1G013200 TROL
7G098200 FD2_B
9G021400 FD3
h 0 1 2 4 1 2 4
FC bins
FC ≥ 5
(5, 2]
(2,0)
FC = 0
(0, -2)
(-5, -2)
FC ≤ -5
Log2(TPM+1)
10
8
6
4
b
Time
h
C
0
HL
1 2 4
HT
1 2 4
Gene ID
Gene Name
SUC
SWEET
1G210500 SUC3
3G426800 SUC4
9G528600
5G225300 SWEET4/5/6/7
9G414000 SWEET16/17
5G408600 SWEET1a
3G333000 SWEET11
3G241200 SWEET1b
3G079200 SWEET3a
5G287700 SWEET2b
0 1 2 4 1 2 4
FC bins
FC ≥ 5
(5, 2]
(2,0)
FC = 0
(0, -2)
(-5, -2)
FC ≤ -5
Log2(TPM+1)
10
8
6
4
2

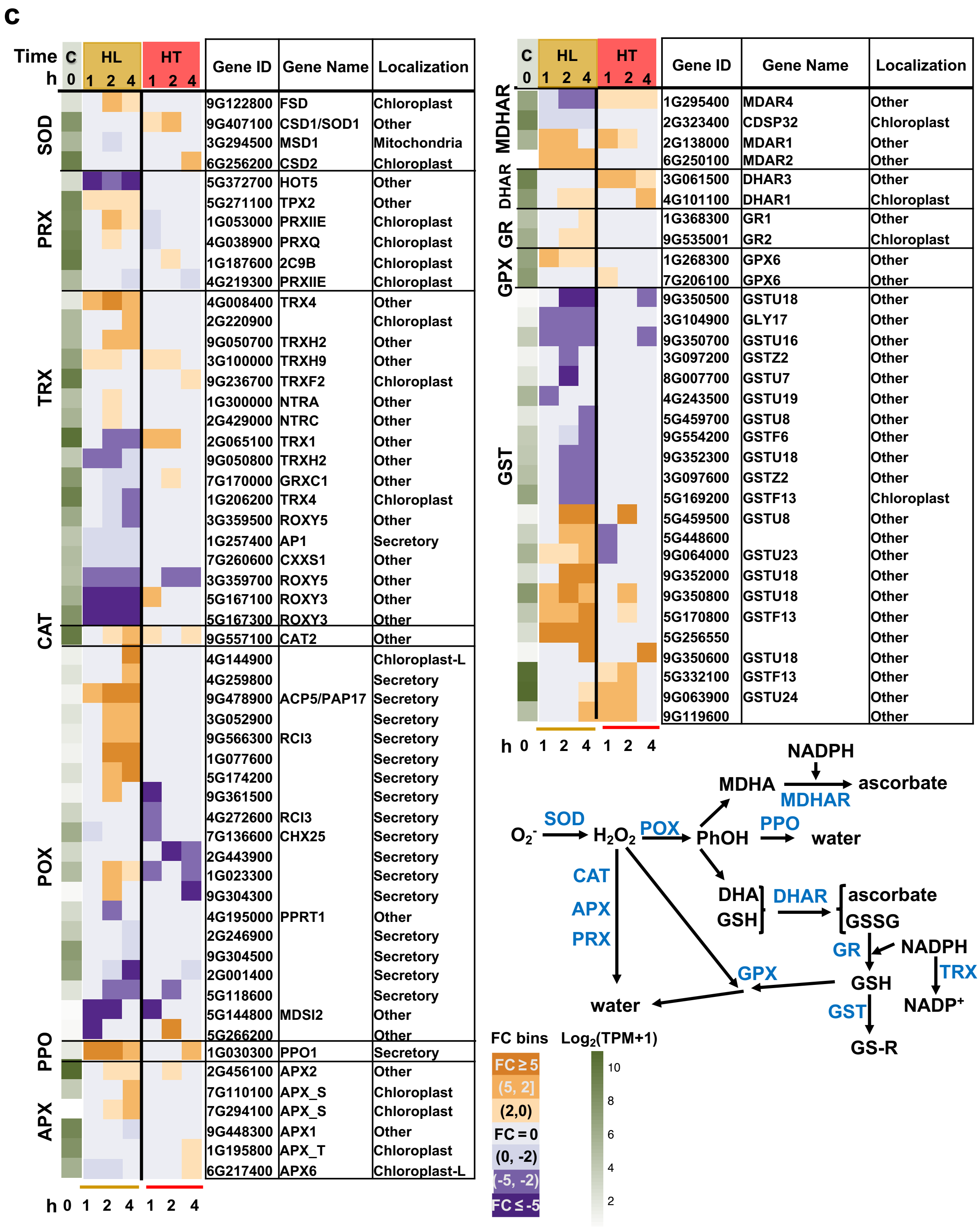

Supplementary Figure 4. High light or high temperature differentially regulated genes involved in various pathways associated with photosynthesis. (a) Alternative light reactions of photosynthesis. *PGR5 (proton gradient regulation 5)* and *PGRL1*

(PGR5-like photosynthetic phenotype 1) are genes involved in cyclic electron transport around PSI. **(b)** Genes encoding sugar transporters. *SUC (Sucrose-proton symporters)* and *SWEET (Sugar Will Eventually Exported Transporters)* encode sucrose transporters. **(c)** Genes involved in antioxidant defense pathways**.** SOD: superoxide dismutase; PRX: peroxiredoxins; TRX: thioredoxin; CAT: catalase. POX: peroxidases. PPO: polyphenol oxidase; APX: ascorbate peroxidase; MDHAR: monodehydroascorbate reductase; DHAR: dehydroascorbate reductase; GR: glutathione reductase; GPX: glutathione peroxidase; GST: glutathione S-transferase. These antioxidant enzymes are colored in blue in the antioxidant defense pathways based on Hasanuzzaman et al, 2020[57]. SOD leads the frontline defense in the antioxidant defense system by converting superoxide anion (O_2^-) into hydrogen peroxide (H_2O_2) which is further detoxified to water (H_2O) by one of these enzymes: POX, CAT, APX, PRX, or GPX. MDHA, monodehydroascorbate; PhOH, phenolic compounds; DHA, dehydroascorbate; GSH, reduced Glutathione; GSSG, oxidized glutathione; R, aliphatic, aromatic, or heterocyclic group; NADPH, nicotinamide adenine dinucleotide phosphate. Most antioxidant enzymes have multiple gene family members in *S. viridis*. The first green column displays $\log_2$(mean TPM + 1) at ctrl_0h (at the start of treatments, C). TPM, transcripts per million, normalized read counts. Heatmap displays the fold change (FC) bin of DeSeq2 model output values at 1, 2, 4 h of high light or high temperature versus control at the same timepoint ($q < 0.05$). FC bins: highly induced: FC ≥ 5; moderately induced: 5 > FC ≥ 2; slightly induced: 2 > FC > 0; not differentially expressed: FC = 0; slightly repressed: 0 > FC > -2; moderately repressed: -2 ≥ FC > -5; highly repressed: FC ≤ -5. Gene ID: *S. viridis* v2.1 gene ID, excluding "Sevir.". All genes presented in the heatmaps were significantly differentially regulated in at least one time point.

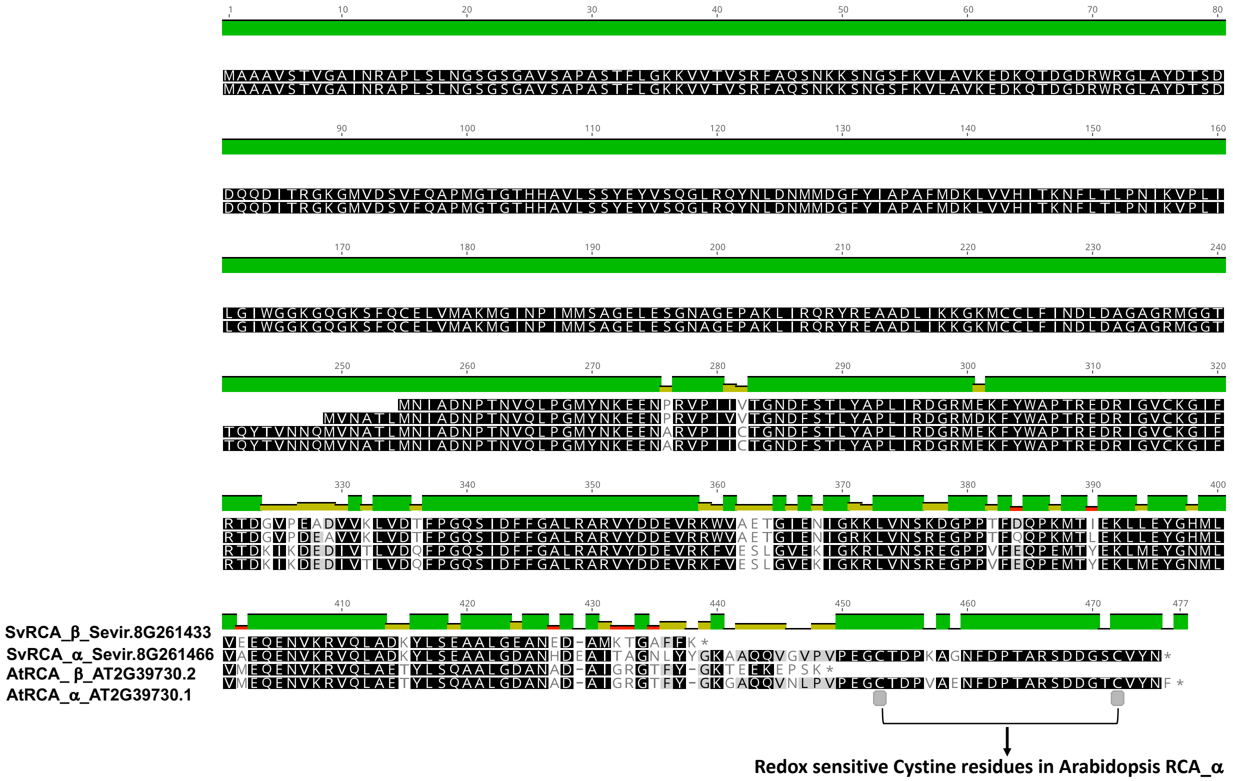

Supplementary Figure 5. Peptide sequence alignment of two *S. viridis* Rubisco Activases (RCAs) with *A. thaliana* RCAs reveals α and β copies of RCA in *S. viridis*. *A. thaliana* RCA_α has two redox sensitive cysteine residues, which are retained in the *S. viridis* RCA_α copy.

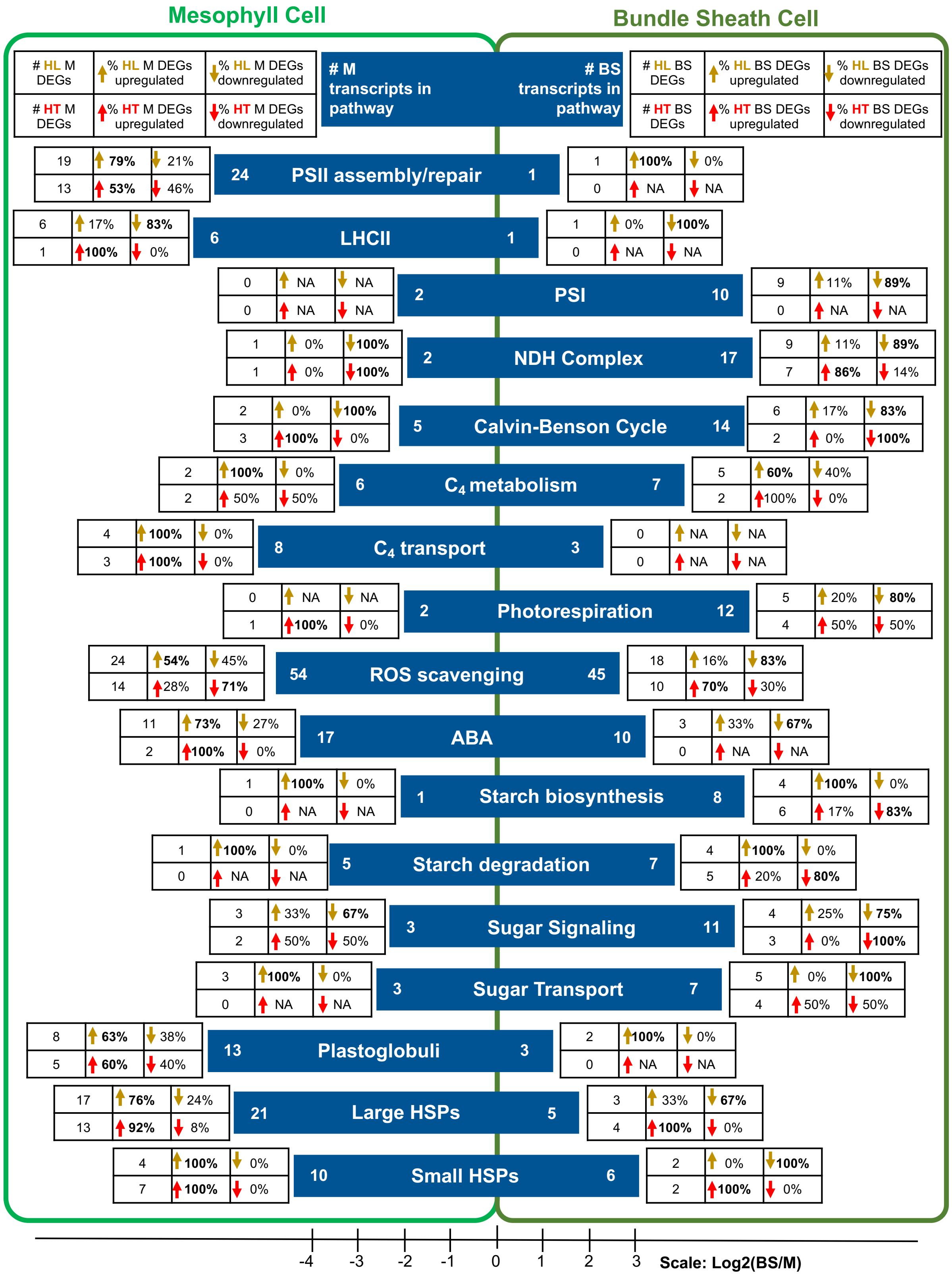

Supplementary Figure 6. Mesophyll (M) and bundle sheath (BS) specificity of

differentially expressed genes reveals cell type specific responses to high light or high temperature. Light and dark green box denotes M and BS cells, respectively. The blue horizontal bars denote pathways of interest we investigated. Position of blue horizontal bars indicates cell type specificity of pathways, and represents the Log2(number of BS specific transcripts/number of M specific transcripts associated with a pathway) according to the published M and BS specific transcriptome data in *S. viridis* under the control condition[58]. Pathways that are preferentially expressed in mesophyll cells have log2(BS/M)<0, e.g. PSII assembly/repair. Pathways that are preferentially expressed in BS cells have log2(BS/M)>0, e.g. PSI. The numbers at the left and right end of each blue horizontal bar represent the numbers of M or BS specific transcripts associated with this pathway. Each pathway has a table of data for each cell type under high light (1st row) or high temperature (2nd row). For each table, the first column indicates the number of M or BS specific transcripts related to a pathway that were differentially expressed in at least one time point. The rest two columns of the table represent the fraction of up- (upward arrows) or down-regulated (downward arrows) transcripts out of the total number of cell-type specific differentially expressed genes (DEGs) related to a pathway. Bolded percentages indicate the larger portions (either up- or down-regulated) in each cell type under high light or high temperature. In high light, 83% of the BS-specific ROS-scavenging DEGs were down-regulated, whereas 54% of M-specific ROS-scavenging DEGs were up-regulated. In contrast, in HT, the majority of BS-specific ROS-scavenging DEGs were up-regulated while M-specific ROS-scavenging DEGs were down-regulated. In high light, all differentially expressed sugar transports were up-regulated in mesophyll cells but down-regulated in BS cells. For HSPs, the majority of M-specific DEGs were up-regulated while the majority of BS-specific DEGs were down-regulated in high light. However, the majority DEGs of HSPs in both M and BS cells were up-regulated in high temperature.

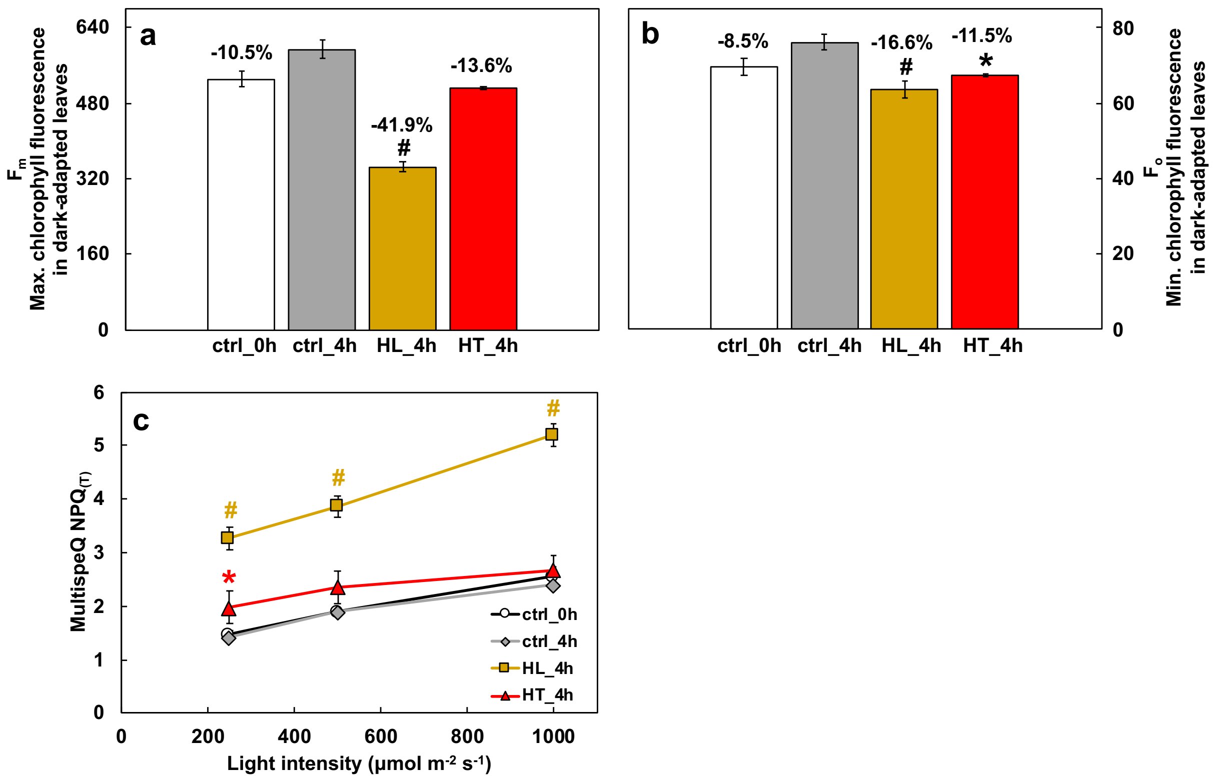

Supplementary Figure 7. High light resulted in significant reduction of maximum chlorophyll fluorescence (F_m) and the HL-induced NPQ was confirmed by using MultispeQ. (a) High light treatments resulted in significantly reduced maximal chlorophyll fluorescence in 20 min dark-adapted leaves (F_m), however, F_m in ctrl_4h leaves were consistent among replicates. **(b)** High light and high temperature treatments resulted in reduced minimum chlorophyll fluorescence in dark-adapted leaves (F_o) but F_o in ctrl_4h leaves were consistent among replicates. Percentages indicate reduction in F_m or F_o compared to ctrl_4h. **(c)** Estimated Non-photochemical quenching, $NPQ_{(T)}$, calculated by F_o' and F_m' obtained in light-adapted leaves using MultispeQ. F_o' and F_m' are minimum and maximum chlorophyll fluorescence in light-adapted leaves. Mean ± SE, *n* = 3-6 biological replicates. Asterisk and pound symbols indicate statistically significant differences of ctrl_0h (at the start of treatments), HL_4h (after 4 h HL), and HT_4h (after 4 h HT) compared to ctrl_4h (after 4 h control treatment) using Student's two-tailed t-test with unequal variance ($*0.01<p<0.05$, $\#p<0.01$). For panel c, the colors of * and # match the significance of the indicated conditions, yellow for HL_4h, red for HT_4h).

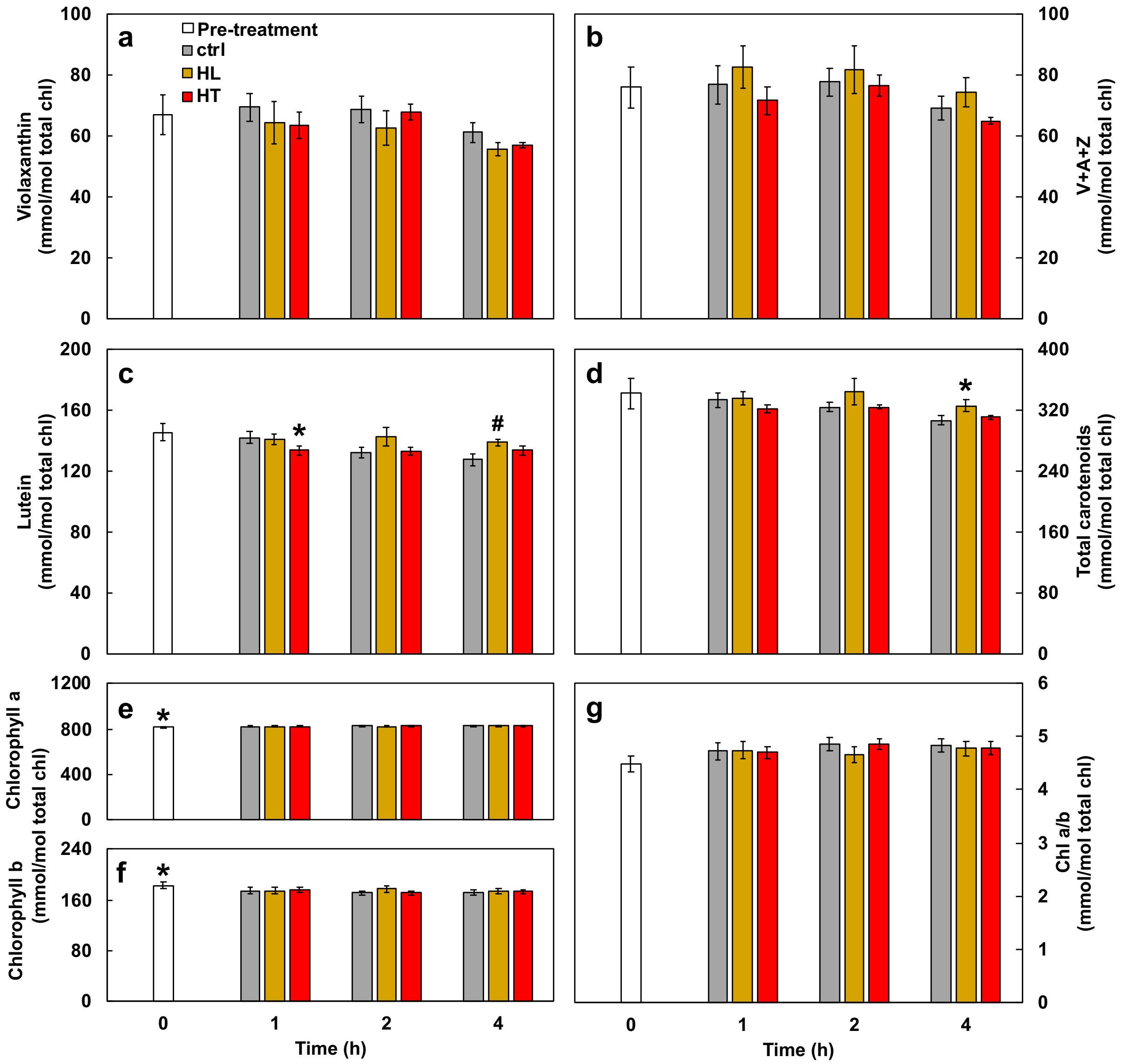

Supplementary Figure 8. High light treatment increased lutein and carotenoids formation. Leaves of *S. viridis* were harvested for high-performance liquid chromatography (HPLC) analysis before treatment or after 1, 2, 4 h treatments of control or high light or high temperature. **(a)** Violaxanthin. **(b)** Total xanthophyll pool (violaxanthin + antheraxanthin + zeaxanthin, V+A+Z). **(c)** Lutein. **(d)** Total carotenoids. **(e,f)** Chlorophyll a and b. **(g)** Chlorophyll *a*/b ratio. Mean ± SE, *n* = 3 biological replicates. *0.01<p<0.05, #p<0.01, compared to control leaves at the same time points. Students' two-tailed t-test with unequal variance.

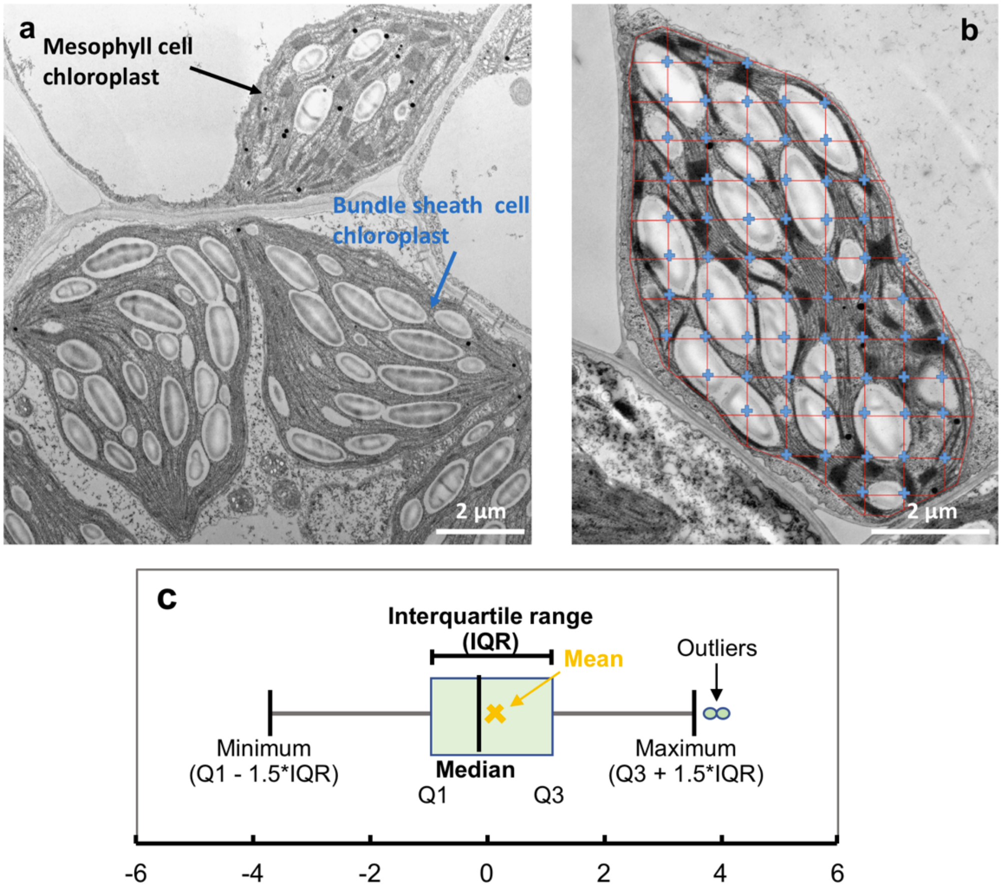

Supplementary Figure 9. Representative transmission electron microscopy (TEM) images illustrate analysis of chloroplast structures. (a) TEM image of chloroplasts of the two cell types in *S. viridis*: mesophyll cells and bundle sheath cells. **(b)** Illustration of Stereo Analyzer analysis for TEM images, which was used to calculate the relative volume of a cellular structures, e.g. starch granules. The Stereo Analyzer outlines a chloroplast with equally spaced uniform grid within the outlined area. The blue crossings of the grid inside the chloroplast are identified as either starch granule, stroma, stroma lamellae, or grana when they overlap with these structures. When all crossings have been identified, the software provides the percentage of relative volume for each structure of interest. **(c)** Illustration of TEM boxplots based on Tukey-style whiskers. Q1, first quartile; Q3, third quartile; IQR, interquartile range. The median value is represented by the vertical black line between Q1 and Q3. The mean value is represented by the yellow X sign.

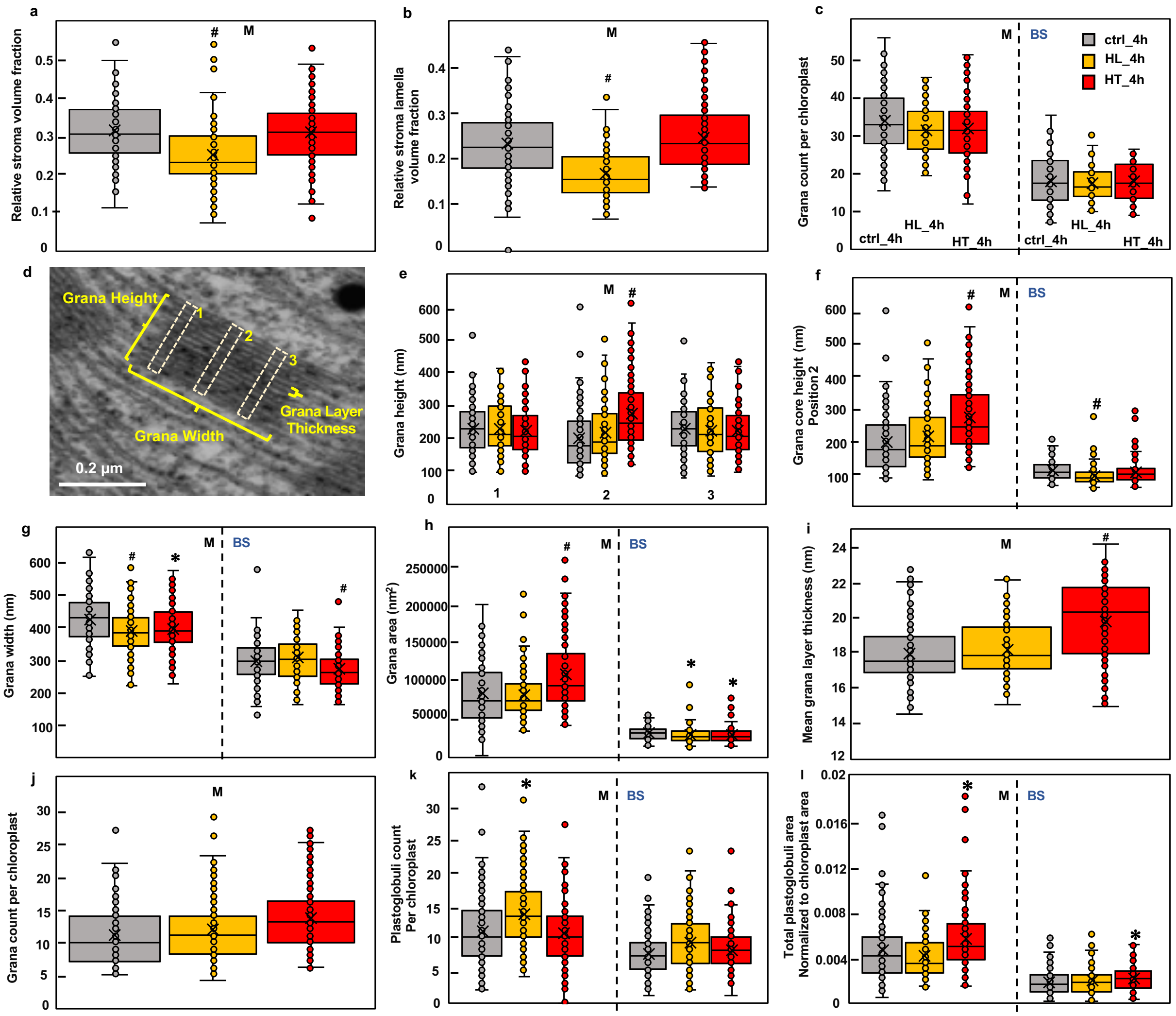

Supplementary Figure 10. High light or high temperature altered various chloroplast structures in *S. viridis* leaves. Chloroplast structure changes after 4 h treatments of control or high light or high temperature. **(a, b)** Relative volume fractions were quantified using Stereo Analyzer with Kolmogorov–Smirnov test for statistical analysis compared to the same cell type of the control condition. **(e, f, g, l)** Parameters related to size and area were quantified using ImageJ with two-tailed t-test with unequal variance compared to the same cell type of the control condition. **(c, j, k)** The counting data was quantified using ImageJ followed by the negative binomial test for significance compared to the same cell type of the control condition. (**d, e**) Position 1 and 3 on grana are to measure the height of grana margin and position 2 is to measure the height of grana core. **(h)** Assuming grana are rectangular, grana area was estimated as grana core

height multiplied by grana width. **(i)** The mean grana layer thickness was calculated as grana core height divided by the number of grana layers. Each treatment had three biological replicates with 90-120 images, *$0.05<p<0.01$; #$p<0.01$.

**All differentially expressed genes (DEGs) sorted into fold change (FC) bins**	**HL_1h**	**HL_2h**	**HL_4h**	**HT_1h**	**HT_2h**	**HT_4h**
Highly induced, FC≥5	176	292	276	87	71	65
Moderately induced, 2≤FC<5	738	1075	949	334	278	316
Slightly induced, 0<FC<2	576	1028	906	382	517	372
No change, FC=0	5301	3731	4261	6537	6468	6597
Slightly repressed, -2<FC<0	534	890	894	341	416	499
Moderately repressed, -5<FC≤-2	616	894	717	398	340	309
Highly repressed, FC≤-5	242	273	180	94	93	25

**Highly induced or repressed genes in at least 1 time point**	**Highly Induced FC≥5**	**Highly Repressed FC≤-5**
**High Light**	496	424
**High Temperature**	167	210
High light & high temperature overlapped	**42**	**13**

Supplementary Table 1. High light had more differentially regulated genes (DEGs) than high temperature while both also had overlapping DEGs. Top: Table of DEGs sorted into bins based on their DeSeq2 fold change (FC) values at each time point of high light or high temperature treatment. Those that are not differentially expressed at a given time point are represented in the “No change, FC = 0” category. Bottom: Number of genes that are highly induced (FC ≥ 5) or highly repressed (FC ≤ -5) in at least one time point in either high light or high temperature treatment.

Key	
Increase	↑
Decrease	↓
Not Available	N/A
Not Significant	N.S.

Table of Parameters Compared to Control Condition

		Treatment Condition:				
		HL_M	HL_BS		HT_M	HT_BS
	Relative Starch Volume	↑ 58%	↑ 67%		↓ 19%	↑ 10%
	Chloroplast Area	↑ 32%	↑ 29%		N.S.	↓ 11%
Chloroplast crowdedness	Relative Stroma + Stroma Lamella Volume	↓ 24%	↓ 36%		N.S.	N.S.
	Relative Stroma Volume	↓ 20%	N/A		N.S.	N/A
	Relative Stroma Lamella Volume	↓ 29%	N/A		N.S.	N/A
Grana	Relative Grana Volume	N.S.	↓ 38%		↑ 24%	↓ 27%
	Grana Core Height, position 2	N.S.	↓ 16%		↑ 36%	N.S.
	Grana Margin Height, position 1, 3	N.S.	N/A		N.S.	N/A
	Grana Width	↓ 9%	N.S.		↓ 6%	↓ 8%
	Grana Area	N.S.	↓ 11%		↑ 29%	↓ 12%
	Mean Grana Layer Thickness	N.S.	N/A		↑ 10%	N/A
	Grana Count per Chloroplast Area	N.S.	N.S.		N.S.	N.S.
	Grana Count Normalized to Chloroplast Area	↓ 30%	↓ 24%		↓ 16%	N.S.
	Grana Layer Count	N.S.	N/A		N.S.	N/A
PG	PG Count per Chloroplast	↑ 27%	N.S.		N.S.	N.S.
	Individual PG Size	↓ 7%	↑ 11%		↑ 37%	↑ 19%
	Total PG Area per Chloroplast	↑ 20%	↑ 39%		↑ 39%	↑ 26%
	PG Area Normalized to Chloroplast Area	N.S.	N.S.		↑ 21%	↑ 7%

Supplementary Table 2. Summary of chloroplast structure changes by using TEM images in leaves after 4 h treatments of high light or high temperature as compared to the control condition. BS, bundle sheath chloroplast; M, mesophyll chloroplast. PG, plastoglobuli. Mean value of each parameter was used for comparison. Yellow highlighted cells and upward arrows denote increased percentages as compared to the control condition. Blue highlighted cells and downward arrows denote decreased percentage as compared to the control condition. Grey highlighted cells and N/A mean data unavailable due to the difficulties to quantify some structures in the bundle sheath chloroplasts. White cells and N.S. mean no significant differences between high light or high temperature as compared to the control treatment.

High light	Parameters	High temperature
↓	Net CO_2 assimilation, after 4 h stress	↓
↓	Stomatal conductance, during stress	↑
↑	Leaf ABA level	No change
Many ↑	ABA pathway transcripts	Little change
↑ M & BS	PG formation in chloroplasts	↑ M & BS
Many ↑	Genes encoding PG localized proteins	Little change
↑ M & BS	Starch accumulation	↑ in BS but ↓ in M
Many ↑	Starch biosynthesis/degradation transcripts	Little change
↑	Chloroplast crowdedness	Little change
↓	ATP synthase activity	Little change
↓	Transcripts involved in light reaction	Little change
Many ↑	Transcripts involved in photoprotection	Little change
↑	Zeaxanthin	Little change
Significant ↑	NPQ	Slightly ↑
↑	Photoinhibition	Little change
↑ slowly	Transcripts of HSPs	↑ quickly
↑	HSFs, different genes ↑ under HL or HT	↑
Little change	Transcripts of RCA-α	↑
Slightly ↓	Transcripts involved in CEF	Slightly ↑
Stable	Overall transcriptional changes	Transient

Supplementary Table 3. Summarized multi-level changes of *S. viridis* in response to 4 h high light or high temperature treatments as compared to the control treatment. Upward arrows denote increase or induction; downward arrows denote decrease or repression. HL, high light; HT, high temperature; ABA, abscisic acid; M, mesophyll chloroplast; BS, bundle sheath chloroplast; PG, plastoglobuli; NPQ, non-photochemical quenching; HSP, heat shock protein; HSF, heat shock transcription factor; RCA, Rubisco activase; CEF, cyclic electron flow around PSI.

**Stage 1: Dark adaptation:** CO_2 400 µmol mol^{-1}			
Light intensity, µmol photons m^{-2} s^{-1}	Time, min	Logs	Measurement frequency, min
0	20	1	
**Stage 2: Light response:** CO_2 400 µmol mol^{-1}			
Light intensity, µmol photons m^{-2} s^{-1}	Time, min	Logs	Measurement frequency, min
50	5	2	2.5
100	5	2	2.5
200	5	2	2.5
400	5	2	2.5
600	5	2	2.5
800	5	2	2.5
1200	5	2	2.5
1500	10	4	2.5
**Stage 3: CO_2 response:** 1500 µmol photons m^{-2} s^{-1}			
[CO_2] reference			
400			
300			
200			
100			
50			
20			
10			
400 x 8			
500			
600			
700			
800			
900			
1000			
1200			
1500			
400			

Supplementary Table 4. LI-6800 protocol for characterizing photosynthetic parameters. Before or after 4 h different treatments, intact *S. viridis* leaves were dark-adapted in LI-6800 leaf chamber for 20 min to measure the maximum PSII efficiency (F_v/F_m), followed by light response experiment from 50 – 1500 µmol photons m^{-2} s^{-1} light and then CO_2 response experiment at 1500 µmol photons m^{-2} s^{-1} light.

Description	Label	Units	Formula
**Net CO_2 assimilation rate**	A_{Net}	µmol m^{-2} s^{-1}	$A = \frac{Flow(CO_{2_R} - CO_{2_S}\left(\frac{1000 - H_2O_R}{1000 - H_2O_S}\right))}{100S}$<br><br>$Flow$: air flow rate (µmol s^{-1})<br>CO_{2_R}: reference cell CO_2 concentration (µmol mol^{-1})<br>CO_{2_S}: sample cell CO_2 concentration (µmol mol^{-1})<br>H_2O_R: reference cell H_2O mole fraction (mmol mol^{-1})<br>H_2O_S: sample cell H_2O mole fraction (mmol mol^{-1})<br>S: leaf area in cm^2
**Transpiration rate**	E	mol m^{-2} s^{-1}	$E = \frac{Flow(H_2O_S - H_2O_S)}{1000S(1000 - H_2O_S)}$
**Stomatal conductance to water vapor**	g_{sw}	mol m^{-2} s^{-1}	$g_{sw} = \frac{2}{\left(\frac{1}{g_{tw}} - \frac{1}{g_{bw}}\right) + \sqrt{(\frac{1}{g_{tw}} - \frac{1}{g_{bw}})^2 + \frac{4K}{(K+1)^2}(2\frac{1}{g_{tw}} - \frac{1}{g_{bw}})\frac{1}{g_{bw}}}}$<br><br>$g_{tw}$: total conductance of the leaf to water vapor (mol m^{-2} s^{-1})<br>g_{bw}: boundary layer conductance to water vapor (mol m^{-2} s^{-1})<br>K: stomatal ratio
**Intercellular CO_2**	C_i	µmol mol^{-1}	$C_i = \frac{\left(g_{tc} - \frac{E}{2}\right) CO_{2_S} - A}{g_{tc} + \frac{E}{2}}$
**Maximal chlorophyll fluorescence, dark-adapted leaves**	F_m		
**Maximal chlorophyll fluorescence, light-adapted leaves**	F_m'		
**Minimal chlorophyll fluorescence, dark-adapted leaves**	F_o		
**Minimal chlorophyll fluorescence, light-adapted leaves**	F_o'		
**Steady state fluorescence**	F_s		
**Variable chlorophyll fluorescence**	F_v		$F_V = F_m - F_o$
**PSII maximum efficiency in dark-adapted leaves**	F_v/F_m		${F_v}/{F_m} = {F_v}/{F_m} = 1 - \frac{F_o}{F_m}$
**Non-photochemical quenching**	NPQ		$NPQ = \frac{(F_m - F_m')}{F_m'}$
**Estimated NPQ**	$NPQ_{(T)}$		$NPQ_{(T)} = \left(\frac{4.88}{\frac{F_m'}{F_o'} - 1}\right) - 1$
**PSII operating efficiency**	ΦPSII		$\Phi PSII = 1 - \frac{F_s}{F_m'}$
**Electron transport rate**	ETR	µmol m^{-2} s^{-1}	$ETR = (\Phi PSII)(0.5)(Qabs_{fs})$<br><br>$Qabs_{fs}$: absorbed light corresponding to the last F_s measurement
**Fraction of open PSII centers**	q_L		$q_L = q_P * \frac{F_o'}{F_s}$
**Plastoquinone redox status**	Q_A		$Q_A = 1 - q_L$

Supplementary Table 5. Formulas to calculate photosynthetic parameters.
