# Supplementary material for: High light and temperature reduce photosynthetic efficiency through different mechanisms in the C4 model Setaria viridis
Source: Commun Biol. 2021 Sep 16;4:1092. doi: 10.1038/s42003-021-02576-2 (PMC8446033; doi:10.1038/s42003-021-02576-2)
Supplement: Supplementary file 3 — Description of Supplementary Files. [file 42003_2021_2576_MOESM3_ESM.pdf]

Description of Additional Supplementary Files

File name: Supplementary Data 1
Description: Normalized read counts in Transcripts Per Million (TPM) for all genes in all time points and biological replicates. Annotation information includes the *S. viridis* provisional defline, *A. thaliana* and *O. sativa* best hits and deflines from the Joint Genome Institute bulk annotation information.

File name: Supplementary Data 2
Description: Differential expression data for all genes that were significantly differentially expressed in at least one time point and condition (DeSeq2, FDR < 0.05). Annotation information includes the *S. viridis* provisional defline, *A. thaliana* and *O. sativa* best hits and deflines from the Joint Genome Institute bulk annotation information.

File name: Supplementary Data 3
Description: Genes up-regulated or down-regulated at all time points in high light or high temperature conditions. Annotation information includes the *S. viridis* provisional defline, *A. thaliana* and *O. sativa* best hits and deflines from the Joint Genome Institute bulk annotation information.

File name: Supplementary Data 4

Description: Overlapping differentially expressed genes between high light and high temperature conditions. Genes differentially expressed in at least one time point were included in lists of up- and down-regulated genes in each condition. Annotation information includes the *S. viridis* provisional defline, *A. thaliana* and *O. sativa* best hits and deflines from the Joint Genome Institute bulk annotation information.

File name: Supplementary Data 5
Description: Genes highly induced or highly repressed (FC ≥ 5, or ≤ -5) in the high light and/or high temperature treatments during at least one time point. Annotation information includes the *S. viridis* provisional defline, *A. thaliana* and *O. sativa* best hits and deflines from the Joint Genome Institute bulk annotation information. Additionally, heat tolerance genes identified in maize with homologs in *S. viridis* are listed. This data file also contains potential gene targets that may help *S. viridis* and other C_4 plants tolerate high light and/or high temperature.

File name: Supplementary Data 6
Description: *S. viridis* v2.1 gene information used to generate heatmaps of pathways of interest.

File name: Supplementary Data 7
Description: Source data for main figures.
